# Mitochondrial Protein Translation: Emerging Roles and Clinical Significance in Disease

**DOI:** 10.3389/fcell.2021.675465

**Published:** 2021-07-01

**Authors:** Fei Wang, Deyu Zhang, Dejiu Zhang, Peifeng Li, Yanyan Gao

**Affiliations:** ^1^Institute for Translational Medicine, The Affiliated Hospital of Qingdao University, College of Medicine, Qingdao University, Qingdao, China; ^2^Key Laboratory of Nuclear Medicine, Ministry of Health, Jiangsu Key Laboratory of Molecular Nuclear Medicine, Jiangsu Institute of Nuclear Medicine, Wuxi, China

**Keywords:** mitochondria, protein translation, translation factors, mitochondrial ribosome, mitoribosome assembly factors, mitochondrial aminoacyl-tRNA synthetase, translation activators, cytoplasmic translation

## Abstract

Mitochondria are one of the most important organelles in cells. Mitochondria are semi-autonomous organelles with their own genetic system, and can independently replicate, transcribe, and translate mitochondrial DNA. Translation initiation, elongation, termination, and recycling of the ribosome are four stages in the process of mitochondrial protein translation. In this process, mitochondrial protein translation factors and translation activators, mitochondrial RNA, and other regulatory factors regulate mitochondrial protein translation. Mitochondrial protein translation abnormalities are associated with a variety of diseases, including cancer, cardiovascular diseases, and nervous system diseases. Mutation or deletion of various mitochondrial protein translation factors and translation activators leads to abnormal mitochondrial protein translation. Mitochondrial tRNAs and mitochondrial ribosomal proteins are essential players during translation and mutations in genes encoding them represent a large fraction of mitochondrial diseases. Moreover, there is crosstalk between mitochondrial protein translation and cytoplasmic translation, and the imbalance between mitochondrial protein translation and cytoplasmic translation can affect some physiological and pathological processes. This review summarizes the regulation of mitochondrial protein translation factors, mitochondrial ribosomal proteins, mitochondrial tRNAs, and mitochondrial aminoacyl-tRNA synthetases (mt-aaRSs) in the mitochondrial protein translation process and its relationship with diseases. The regulation of mitochondrial protein translation and cytoplasmic translation in multiple diseases is also summarized.

## Introduction

Mitochondria are important, semi-autonomous organelles in eukaryotic cells. Their independent genetic system includes mitochondrial DNA (mtDNA), messenger RNA (mRNA), transfer RNA (tRNA), ribosomal RNA (rRNA), and ribosomes. Mitochondrial DNA replication, mRNA transcription, and protein translation occur independently, including the synthesis of polypeptides encoded by mtDNA. Human mtDNA comprises 16,569 base pairs and exists in multiple copies (Surovtseva et al., 2011). The mitochondrial genome lacks introns and contains only one major non-coding region, as well as a displacement loop (D-loop), which contains the promoter for transcription initiation. Mitochondrial DNA has heavy (H) and light (L) strands. The L-chain is rich in adenine and thymine, encoding one protein (ND6) and eight tRNAs. The H-strand is rich in guanine and encodes the remaining 12 proteins and 14 tRNAs (Shokolenko and Alexeyev, 2015). Despite the presence of this system, most mitochondrial proteins are encoded by nuclear DNA (nDNA). Only 13 of the proteins involved in the respiratory chain of mitochondria are encoded by mtDNA. These 13 are the core components of the mitochondrial respiratory chain complex. ND1–ND6 and ND4L are subunits of complex I. Cytochrome b (CYTB) is a subunit of complex III. COX1–COX3 are subunits of complex IV. Finally, ATP6 and ATP8 are subunits of ATPase (complex V). All complex II subunits are encoded by nDNA and are translocated through the mitochondrial membrane (Schulz et al., 2015).

**FIGURE 1:**
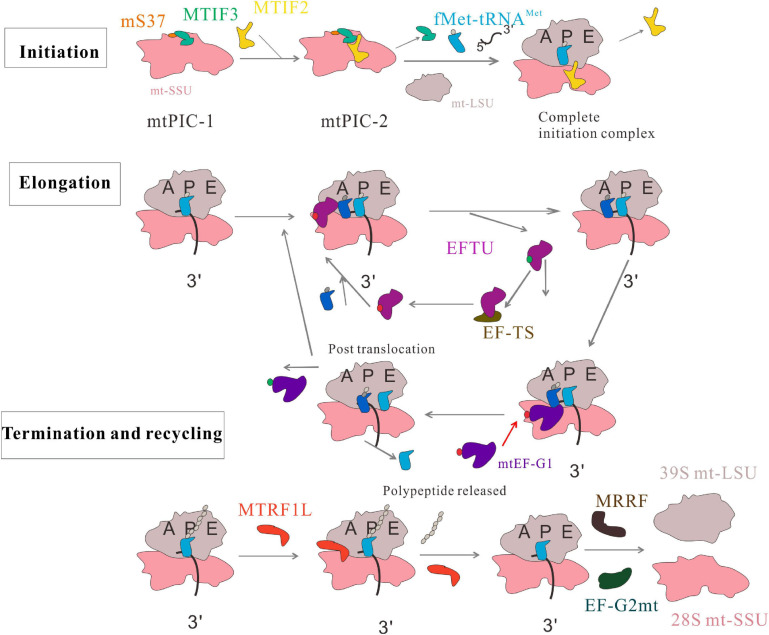
Human mitochondrial translation. The process includes four phases: initiation, elongation, termination, and recycling. In the initiation phase, two distinct pre-initiation assembly steps, termed mitochondrial preinitiation steps 1 and 2 (mtPIC-1, mtPIC-2) are established. In the elongation phase, the aminoacyl-tRNA is transferred to the A site of mitochondrial ribosome by GTP ⋅ EFTU, and GTP ⋅ EFTU is transformed into GDP ⋅ EFTU. EF-TS converts GDP ⋅ EFTU to GTP ⋅ EFTU. The peptide-tRNA in the P site is transferred from the P site to the A site. mtEF-G1 binds to the ribosome at the A site and promotes translocation of the ribosome along the mRNA by inducing movement of A-tRNAs and P-tRNAs to the P site and E site. The tRNA at the E site leaves the monomer and this cycle continues until the polypeptide is completed and the stop codon appears at the A site. MRRF and EF-G2mt promote the separation of ribosomal subunits; MTIF3 combines with the small mitochondrial ribosomal subunits to prevent the premature reassociation of the large and small subunits.

Mitochondrial translation includes initiation, elongation, termination, and ribosome-recycling stages (Figure 1). The mtDNA encodes two rRNAs, 22 tRNAs, and 13 proteins, which are important for mitochondrial function and biogenesis (Yokokawa et al., 2018). Mammalian mitochondrial ribosomes (mitoribosomes) synthesize proteins essential for ATP production via oxidative phosphorylation (OXPHOS). Mitochondrial translation mechanisms differ from those of cytoplasmic ribosomes, and are more similar to prokaryotic translation (Koripella et al., 2019a). At each stage, mitochondrial protein synthesis requires a series of mitochondrial factors (Mai et al., 2017). These include two initiation factors (MTIF2 and MTIF3), three elongation factors (EFTU, EF-TS, and mtEF-G1), a release factor (MTRF1L), and two ribosome recycling factors (MRRF and EF-G2mt). All are necessary for mitochondrial translation. Deficiency or mutation of these factors leads to abnormal mitochondrial translation and a series of metabolic disorders.

Translation in mitochondria is carried out by specialized mitoribosomes. Mammalian mitochondrial ribosomes consist of a large subunit (39S) and a small subunit (28S). The large subunit contains a 16S rRNA and 48 proteins, while the small subunit contains a 12S rRNA and 30 proteins. To visualize the process of translation in human mitochondria, Aibara et al. (2020) determined eight cryogenic electron microscopy (cryo-EM) structures of human mitoribosomes in complex with mitochondrial mRNA (mt-mRNA), mitochondrial tRNAs (mt-tRNAs) and additional factors in different states. Most mutations in nuclear genes encoding mitoribosomal proteins, as well as mitochondrial DNA encoding tRNAs and 12S rRNA, lead to clinically and genetically heterogeneous infant multisystem diseases, such as Leigh’s syndrome, sensorineural hearing loss, encephalomyopathy, and hypertrophic cardiomyopathy (Jacobs and Turnbull, 2005; Perez-Martinez et al., 2008; Rotig, 2011).

Mitochondria are involved in many physiological and pathological processes, including electron transport, OXPHOS, fatty acid metabolism, and the tricarboxylic acid cycle. Changes in mitochondrial protein structure and function are involved in many human diseases, including nervous diseases, cardiovascular diseases, and cancers (De Silva et al., 2015; Mai et al., 2017; Ferrari et al., 2020; Kummer and Ban, 2021). Most mitochondrial diseases are characterized by oxygen- and phosphorus-related damage, affecting the OXPHOS system (Torraco et al., 2015). Many mitochondrial diseases are caused by defective mitochondrial protein synthesis. Mutations or deficiencies in mitochondrial protein translation factors (Table 1), mitochondrial tRNAs, mitochondrial aminoacyl-tRNA synthetases (mt-aaRSs), mitochondrial ribosomal proteins (MRPs), or mRNA, rRNA, and tRNA modification enzymes can lead to translation disorders and a wide range of phenotypes and diseases.

**TABLE 1:** Mitochondrial translation factor mutations and related diseases.

**Gene**	**Protein**	**Related disease/clinical presentation**	**References**
**(1) Mitochondrial translation initiation process**			
***MTIF2***	**MTIF2**	Pathological myocardial hypertrophy	Lee et al., 2019
***MTIF3***	**MTIF3**	Parkinson’s disease	Behrouz et al., 2010
		Obesity	Abadi et al., 2016
		Cardiomyopathy	Rudler et al., 2019
**(2) Mitochondrial translation elongation process**			
***TUFM***	**EFTU**	Lactic acidosis and fatal encephalopathy	Cao and Qin, 2016
		Lung cancer, colorectal carcinoma	Shi et al., 2012; He et al., 2016; Xu et al., 2019
		Hyperlactatemia	Di Nottia et al., 2017
		Metabolic cardiomyopathy	Hershkovitz et al., 2019
		MELAS	Sasarman et al., 2008
		Myocardial ischemia and reperfusion	He et al., 2001
		Polycystic encephalopathy, micropolygyria	Valente et al., 2009
***TSFM***	**EF-TS**	Early onset encephalocardiomyopathy	Emperador et al., 2016
		Mitochondrial cardiomyopathy	Perli et al., 2019
		Hypertrophic or dilated cardiomyopathy	Seo et al., 2019
		Encephalomyopathy and hypertrophic cardiomyopathy	Smeitink et al., 2006
		Infant liver failure	Vedrenne et al., 2012
***GFM1***	**mtEF-G1**	Early onset Leigh syndrome	Valente et al., 2007
		MELAS	Ahola et al., 2014; Emperador et al., 2016
***GUF1***	**mtEF4**	Cancer	Zhu et al., 2018
		Western syndrome	Alfaiz et al., 2016
		Male infertility	Gao et al., 2016
		Peripheral neuropathy, spastic paraparesis	Temperley et al., 2003
		Axonal neuropathy and optic atrophy	Tucci et al., 2014
		Distal motor neuropathy, optic atrophy	Fang et al., 2017b
		Leigh syndrome	Imagawa et al., 2016
		Leigh syndrome, optic atrophy, ophthalmoplegia	Antonicka et al., 2010
		Classical Behr’s syndrome phenotype	Pyle et al., 2014
		Optic atrophy and mild developmental delays	Heidary et al., 2014
		Spastic paraplegia and strabismus	Buchert et al., 2013
**(3) Mitochondrial termination and ribosome recycling**			
***MRRF***	***RRF***	Parkinson’s disease	Wu et al., 2020
***GFM2***	**EF-G2mt**	Leigh syndrome with arthrogryposis multiplex congenital	Fukumura et al., 2015
***MTRFR***	**C12orf65**	Early onset optic atrophy, progressive encephalomyopathy	Fang et al., 2017a
**(4) Mitochondrial translational activators and disease**			
***LRPPRC***	**LRPPRC**	French-Canadian Leigh syndrome	Kohler et al., 2015
**(5) Mitochondrial miRNA**			
**miR-181c**		Heart failure	Das et al., 2012, 2014
**miR-1**		Myogenesis	Das et al., 2014
**miR-92a**		Diabetic cardiomyopathy	Li et al., 2019
**miR-21**		Myocardial hypertrophy	Li et al., 2016

## Abnormal Mitochondrial Translation Initiation and Disease

The translation initiation process is a highly regulated and rate-limiting step in mitochondrial protein synthesis, which begins with the formation of an initiation complex. In bacteria, the first step of translation initiation is the dissociation of the ribosome into its small subunit (SSU) and large subunit (LSU). Then in the presence of mRNA, formylated methionine (fMet)-tRNA^Met^ (fMet-tRNA^Met^), and three initiation factors (IF1, IF2, and IF3), the initiation complex can be formed to initiate protein synthesis. In mammalian mitochondria, the separation of the 28S and 39S mitochondrial ribosomal subunits leads to the formation of an initiation complex consisting of the 28S subunit, mRNA, fMet-tRNA^Met^, and initiation factors (Kuzmenko et al., 2014). These initiation factors (MTIFs) are encoded by nuclear genes that regulate the initiation of mitochondrial translation. One of these, MTIF2, closes the decoding center and stabilizes the binding of fMet-tRNA^Met^ to leaderless mRNAs (Rudler et al., 2019). Therefore, MTIF2 promotes the binding of fMet-tRNA^Met^ with the SSU of mitoribosomes (Gaur et al., 2008). Another important initiation factor is MTIF3, whose function has been widely studied. It enables the initiation codon (AUG) to correctly localize to the peptide (P) site of the mitoribosome and helps the mRNA bind to the mitochondrial SSU. In the absence of mRNA, fMet-tRNA^Met^ and MTIF2 bind weakly to mitochondrial SSU; MTIF3 acts to prevent or correct the premature binding of these components (Christian and Spremulli, 2009). Furthermore, a recent paper mentioned two distinct pre-initiation assembly steps, termed mitochondrial preinitiation steps 1 and 2 (mtPIC-1, mtPIC-2). The study applied cryo-EM and fluorescence analysis to reveal that the interaction between mitochondrial-specific protein mS37 and MTIF3 keeps the mitochondrial SSU in a conformation favorable for the accommodation of MTIF2 in the second step. Then, MTIF2 produces an intermediate state mtPIC-2, which binds to the mitochondrial LSU, replaces MTIF3 with the initial tRNA, and accommodates mitochondrial leaderless mRNA, resulting in the formation of a complete elongation-competent initiation complex (Khawaja et al., 2020) (Figure 1). Some diseases are associated with abnormal mitochondrial translation initiation and mutations in the genes encoding translation initiation factors.

### MTIF2

Despite its role in mitochondrial translation, MTIF2 is encoded by a nuclear gene. It participates in the activation of mitochondrial protein translation and is the main regulatory factor for initiation. It is considered to be the functional equivalent of bacterial IF1 and IF2 (Liao and Spremulli, 1990). A 37-residue sequence was inserted into the V and VI domains of MTIF2, making it substitute for the function of IF1. A cryo-EM study showed that this 37 amino acid insertion into MTIF2 produced a similar function to that of IF1, which could stereoscopically block the ribosomal A site, thus promoting the binding of the initial tRNA to the ribosomal P site during translation initiation (Yassin et al., 2011). This insert interacts with the decoding center of the small ribosomal subunit A-site and the 3′-CCA end of the sarcin/ricin loop (SRL) and fMet-tRNA^Met^ near the large ribosome subunit peptidyltransferase center (PTC), under the action of guanosine triphosphate (GTP) and the mRNA template to generate a pre-start complex (Gaur et al., 2008). Mutation of this insertion domain seriously affects the ability of MTIF2 to bind to mitochondrial SSU and the formation of initiation complexes (Spencer and Spremulli, 2005). Similar to other protein synthesis systems, mitochondrial translation is initiated by methionine residues. However, for mitochondria, only one type of tRNA^Met^ is used, which is in the form of fMet-tRNA^Met^ at initiation and Met-tRNA^Met^ during elongation. Formylation of Met-tRNA^Met^ markedly enhances its affinity for MTIF2 (Spencer and Spremulli, 2004; Kummer et al., 2018). MTIF2 contains domains III to VI which are homologous to *Escherichia coli* IF2. Of these, subunit IV is the domain that mainly binds to fMet-tRNA^Met^ (Spencer and Spremulli, 2004). Subsequently, Kummer et al. (2018) analyzed the cryo-EM structure of the complete translation initiation complex of mammalian mitochondria. They showed that the function of the additional domain insertion of MTIF2, which stabilizes the binding of leaderless mRNAs by closing the decoding center, induces the conformational changes of rRNA nucleotides involved in decoding. H678 of MTIF2 domain IV interacts with formyl of fMet-tRNA^Met^, while F632 interacts with methionine. The results showed that MTIF2 has a unique function in the identification of fMet-tRNA^Met^ and the regulation of GTPase activity (Kummer et al., 2018). Thus, MTIF2 has dual functions in mammalian mitochondria (Gaur et al., 2008). Therefore, mtIF2 guides the association of fMet-tRNA^Met^ with mRNA and the assembly of mitochondrial 55S ribosomes (D’Souza and Minczuk, 2018).

Previous studies have found a relationship between MTIF2 and cardiomyocyte death (Lee et al., 2019), but no mutation of the *MTIF2* gene leading to mitochondrial diseases has been found. Specifically, in cell and animal models, MTIF2 has been linked to the oxidative capacity and redox state of cardiomyocytes (Lee et al., 2019). Its expression is reduced in the hearts of aged and obese mice, which decreases the oxidative capacity of cardiomyocytes. After *in vitro* hypoxic exposure, insufficient expression of MTIF2 can reduce oxygen consumption and increase cardiomyocyte death. Therefore, MTIF2 is necessary to maintain the oxidative properties of the myocardium, which plays a role in pathological myocardial hypertrophy during aging and obesity (Lee et al., 2019). The lack of MTIF2 in *Saccharomyces cerevisiae* reportedly results in disordered mitochondrial protein synthesis, which affects respiration. This defect was restored by either the *S. cerevisiae MTIF2* gene (*IFM1*) or cDNA encoding bovine MTIF2 (Tibbetts et al., 2003). A recent study showed that the upregulation of MTIF2 is associated with poor prognosis of lung cell malignancy induced by inorganic arsenic (Zhang et al., 2019).

### MTIF3

Mammalian MTIF3 is encoded by nuclear genes. It catalyzes the formation of initiation complexes in the presence of mitoribosomes, mRNA, mitochondrial initiator tRNA, and MTIF2 *in vitro*, and regulates mitochondrial protein translation. It contains N-terminal and C-terminal domains (NTD and CTD, respectively) that are separated by unstructured, flexible connection zones (Koc and Spremulli, 2002). Unlike its bacterial homolog, MTIF3 possesses unique terminal extensions on its N- and C-termini (Next and Cext, respectively). These extensions may have evolved as adaptations to the mitochondrial environment (Chicherin et al., 2019). The CTD participates in translation regulation *in vitro* (Haque and Spremulli, 2008). It may also play a role in the kinetics of translation initiation complex formation (Haque and Spremulli, 2008). One finding also showed that the NTD increases the fidelity function of MTIF3 in terms of the selection of the initiation codon and initiator tRNA (through its anticodon stem) (Ayyub et al., 2017). Koripella et al. (2019a) analyzed the cryo-EM structure of the mammalian mitochondrial 28S-MTIF3 complex. Unique contacts between the N-terminal domain (NTD) of MTIF3 and the 28S subunit were observed in the cryo-EM structure, which also explained the high affinity of MTIF3 for the 28S subunit. The location of mito-specific N-terminal extensions (NTE) of MTIF3 indicated the role of NTE in binding of the initial tRNA to the 28S subunit. The location of the CTD imparts anti-association activity, and the orientation of mito-specific C-terminal extensions (CTEs) explains why it can destabilize initiator tRNA in the absence of mRNA. The authors also speculate that CTD can recruit leaderless mRNAs and initiate translation. The study investigated the role of the NTD and CTD of MTIF3 in stabilization of the pre-initiator complex with mitochondrial SSU, also demonstrating the mutual binding site of MTIF3 and tRNA on the ribosome (Koripella et al., 2019a).

Mammalian MTIF3 has an affinity for small mitoribosome subunits. It locates the AUG or AUA promoter of mRNA at the P-site of 28S SSU, prevents the premature binding of 39S large subunits with the 28S SSU, and promotes the dissociation of mitoribosomes (55S) into small (28S) and large (39S) ribosomal subunits (Koc and Spremulli, 2002). It changes the equilibrium between the 55S mitoribosome and the separated 39S and 28S subunits by binding with free 28S, preventing further subunit rebinding. In addition, MTIF3 binds to the 55S mitoribosome and promotes its dissociation. This may be due to the formation of a transient intermediate that is rapidly distributed to the 28S subunit combined with MTIF3 and the free 39S subunit (Christian and Spremulli, 2009). Moreover, mammalian MTIF3 promotes the separation of the initiator tRNA from mitoribosomes with a lack of mRNA (Bhargava and Spremulli, 2005).

Another key role of MTIF3 is in mitochondrial translation initiation by regulating mitochondrial function. One study indicated that MTIF3 is essential for mitochondrial translation initiation and the coordinated assembly of respiratory complexes. Heart-specific and skeletal muscle-specific loss of MTIF3 in mice caused abnormal mitochondrial protein synthesis and induced cardiomyopathy (Rudler et al., 2019). Changes in the function or expression of MTIF3 protein may affect mitochondrial function, ATP production, or the formation of reactive oxygen species (ROS), affecting susceptibility to Parkinson’s disease (PD) and promoting its occurrence (Behrouz et al., 2010). The latter investigators also described an association of the rs7669 variant of *MTIF3* with PD risk. Whether rs7669 is a functional variant remains to be confirmed. Thus, *MTIF3* mutations are associated with multiple pathological processes such as PD, obesity, and diabetes. A synonymous polymorphism (Asp266Asp, caused by c.798C>T) is associated with sporadic PD, indicating that MTIF3 may be involved in the pathogenesis of PD (Anvret et al., 2010), and this single-nucleotide polymorphism (SNP, c.798C>T) in *MTIF3* was shown to be strongly related to the PD allele by another group (Abahuni et al., 2007). MTIF3 is also involved in obesity (Abadi et al., 2016). It was reported that an *MTIF3* SNP (rs4771122) is associated with increased body mass index (BMI) in Mexican children (Abadi et al., 2016). In addition, MTIF3 autoantibodies were found in patients with type I diabetes, indicating that MTIF3 is related to diabetes (Bian et al., 2017).

## Mitochondrial Translation Elongation and Disease

Elongation is the core of protein synthesis and is the most conserved (Ott et al., 2016). In the process of protein synthesis, ribosomes move along the mRNA, which is decoded continuously through the interaction between mRNA codons and the anticodons of cognate tRNAs on the SSU. The aminoacyl (A) site, the peptidyl (P) site, and the exit (E) site are three binding sites for tRNAs in the ribosome. EFTU, EF-TS, mtEF-G1, and GUF1 are involved in the regulation of mitochondrial translation elongation. EFTU brings aminoacyl-tRNA (aa-tRNA) to the ribosomal A-site and coordinates specific codon: anticodon pairing between the mRNA and tRNA. Then, EFTU mediates hydrolysis of GTP and release of newly formed EFTU ^⋅^ guanosine diphosphate (GDP). EF-TS promotes GDP release from EFTU. GDP, regenerating EFTU ^⋅^ GTP. Next, under the catalysis of the large ribosomal subunit, peptide bonds are formed between the peptide-tRNA at the P site and aa-tRNA at the A site. mtEF-G1 (also called GFM1) then binds to the ribosomal A site and promotes ribosomal translocation along the mRNA by inducing A- and P-tRNAs to move to the P and E sites, respectively (Christian and Spremulli, 2012; Hallberg and Larsson, 2014; Ott et al., 2016) (Figure 1). The clinical expression levels of EFTU, EF-TS, and mtEF-G1 can reflect the function and translational speed of mitochondria; they can be used to evaluate the functional state of cells. Mutations in genes encoding these factors lead to mitochondrial disease (Wang X. Q. et al., 2017). Clinical manifestations include diverse diseases and phenotypes. These are usually disabling, progressive, and/or fatal, affecting the brain, liver, skeletal muscle, heart, and other organs (Boczonadi and Horvath, 2014; Diodato et al., 2014).

### EFTU

A highly conserved GTPase, EFTU is encoded by the *TUFM* gene. EFTU is highly conserved and has 55–60% homology with bacterial EFTU (Woriax et al., 1995). In its active form (EFTU ^⋅^ GTP), aa-tRNA is transferred to the A-site of the mitoribosome to coordinate codon:anticodon pairing between the mRNA and tRNA through the formation of a ternary complex. Because this process requires energy, EFTU ^⋅^ GTP is converted to the EFTU ^⋅^ GDP inactive complex by EFTU-mediated GTP hydrolysis. The latter is released from ribosomes as the substrate of EF-TS, which promotes the exchange of GDP and GTP, reactivating GTP (Di Nottia et al., 2017). After correct codon:anticodon pairing, EFTU ^⋅^ GDP leaves the mitoribosome and aa-tRNA enters the P-site. At the P-site, the formation of peptide bonds is catalyzed by the large ribosomal subunit and the growing peptide chain is elongated (Boczonadi and Horvath, 2014). Binding at the A-site, GFM1 promotes the translocation of the ribosome along the mRNA by inducing movement of A-tRNAs and P-tRNAs to the P site and E site, respectively (Cao and Qin, 2016). The overall structure of EFTU ^⋅^ GTP is similar to that observed in *E. coli*, but the nucleotide binding domain (domain I) is in a different orientation compared with that observed in prokaryotic EFTU. In addition, domain III is followed by a short extension of 11 amino acids, forming one helical turn (Polekhina et al., 1996). It is an important step for EFTU to select the correct aa-tRNA for the ribosome A site to ensure the fidelity of translation. Using structure-based and explicit solvent molecular dynamics simulations based on recent cryo-EM reconstructions, Girodat et al. (2020) investigated the structural mechanism of how EFTU is involved in proofreading. They found that switch I of EFTU is a gate that facilitates aa-tRNA selection. Switch I of EFTU converts from an α-helix to a β-hairpin to control the movement of aa-tRNA in the accommodation corridor through steric interactions between Arg58 and the correct acceptor stem of aa-tRNA. Recent studies have also shown that EFTU plays a non-canonical role in the regulation of mitophagy mediated by PINK1. EFTU has mitochondrial-cytosolic dual localization. Ser222 of EFTU is phosphorylated by PINK1, which localizes it mainly in the cytosol and plays a role in inhibiting mitophagy (Lin et al., 2020).

Mutations in *TUFM* are associated with OXPHOS deficiency, which leads to lactic acidosis and fatal encephalopathy (Cao and Qin, 2016). A study described a patient with a homozygous mutation in *TUFM*. The patient was affected by neonatal lactic acidosis, rapidly progressive encephalopathy due to mitochondrial translation disorder, and mtDNA-related mitochondrial respiratory chain (MRC) complex deficiency (Valente et al., 2007). Another study described the case of a female infant with polycystic encephalopathy, micropolygyria, and leukodystrophy changes. An Arg336Gln substitution in EFTU was identified and associated with the failure to form active EFTU; the GTP⋅aa-tRNA ternary complex affected mitochondrial translation (Valente et al., 2009). In mitochondrial encephalomyopathy, lactic acidosis, and stroke-like episodes (MELAS) myoblasts, an almost complete lack of respiratory chain complexes I, IV, and V has been attributed to a heteroplasmic m.3243A>G substitution in the mitochondrial tRNA^Leu^(UUR) (Emperador et al., 2016). The authors reported that the overexpression of EFTU or EF-G2mt (also called GFM2), but not EF-TS or GFM1, partially suppressed the phenotype. The finding of EFTU phosphorylation during myocardial ischemia and reperfusion prompted the hypothesis that the phosphorylation of mitochondrial translation factors inhibits mitochondrial protein synthesis, indicating that mitochondrial protein synthesis is a decisive factor in myocardial ischemia-reperfusion injury (He et al., 2001). Therefore, *TUFM* may affect the function of the mitochondrial respiratory chain by regulating mitochondrial translation and may play an important role in encephalopathy and other diseases.

A novel c.964G>A mutation in *TUFM* changed the evolutionarily conserved EFTU-Gly322 residue to Arg, resulting in an approximately 80% decrease in expression. Patients harboring this pathological variant of *TUFM* displayed metabolic acidosis and hyperlactatemia. Neurological examination revealed severe encephalopathy and leukodystrophy with microlymph nodes (Di Nottia et al., 2017). The cases presented in these studies have expanded the phenotypic characteristics of *TUFM*-related diseases, which are characterized by lactic acidosis and dilated cardiomyopathy, without progressive encephalopathy. These findings have implicated *TUFM* as a candidate gene for early cardiomyopathy and in the differential diagnosis of metabolic cardiomyopathy (Hershkovitz et al., 2019).

Moreover, some studies have shown that EFTU is involved in the process of epithelial-to-mesenchymal transition (EMT); thus, it is expected to become a new prognostic indicator. In human cancer tissues, EFTU has been reported to be downregulated; moreover, EFTU knockdown induced EMT by activating the AMPK-GSK3β/β-catenin pathway (He et al., 2016). Overexpression of EFTU in colorectal carcinoma (CRC) has been described. This may be a promising new prognostic indicator for CRC (Shi et al., 2012). Another study showed that EFTU was deubiquitinated by ubiquitin-specific peptidase 5 (USP5), and its level increased in CRC (Xu et al., 2019). Moreover, EFTU knockout decreased mitochondrial respiratory chain activity, increased glycolysis, and produced ROS, inducing EMT (Samec et al., 2018). However, it has also been reported that high expression of EFTU in gastrointestinal stromal tumors (GISTs) is related to the occurrence, development, and prognosis of the tumors (Weng et al., 2020).

Some studies have shown that EFTU also participates in the process of disease by regulating oxidative stress. Silencing *TUFM* in *Paracoccidioides brasiliensis* reportedly alters translation elongation, causes respiratory defects, and increases the sensitivity of yeast cells to reactive oxygen stress, indicating the involvement of *TUFM* in the pathogenicity of this fungus (Marcos et al., 2019). Moreover, EFTU can physically interact with Xeroderma pigmentosum group D (XPD) protein, which is involved in mitochondrial oxidative DNA damage repair (Liu et al., 2015). Recently published articles have reported that EFTU is associated with Alzheimer’s disease (AD)-like pathologies. The expression of EFTU is decreased in the brains of AD patients. Further studies showed that EFTU participates in the pathological process of AD through ROS in Beta-secretase 1 (BACE1) translation, apoptosis, and tau phosphorylation (Zhong et al., 2021).

### EF-TS

EF-TS is a guanine nucleotide exchange factor encoded by *TSFM*. This factor combines with the EFTU ⋅ GDP complex to promote GDP release and form a stable EFTU ⋅ EF-TS heterodimer. GTP then promotes the separation of EF-TS from this complex and regenerates EFTU ⋅ GTP, which then binds to another aa-tRNA. This ternary complex combines with the ribosome A site. When the correct codon:anticodon recognition is established, GTP is hydrolyzed, EFTU ⋅ GDP is released, and the cycle repeats (Cai et al., 2000; Mai et al., 2017). The three-dimensional crystal structure of the bovine EFTU ⋅ EF-TS complex has been determined (Jeppesen et al., 2005).

Studies have described that decreased EF-TS in fibroblasts induced the upregulation of EFTU and mitochondrial biogenesis-related genes, along with increased expression of respiratory chain subunits and an increase in normal oxygen consumption rate (Perli et al., 2019). Forced overexpression of EFTU in cells obtained from carriers of pathogenic *TUFM* mutations can rescue EFTU deficiency. The predicted instability of EF-TS and EFTU in a bioinformatics analysis was consistent with a significant decrease in the steady-state levels of both proteins in clinically affected myocardium (Perli et al., 2019). These findings indicate that the lack of respiratory chain enzymes leads to OXPHOS dysfunction and eventually to multiple mitochondrial diseases.

Symptoms of mitochondrial cardiomyopathy caused by mitochondrial translation disorder have been described for a patient with a new complex heterozygote variant in *TSFM* (Perli et al., 2019). Two novel compound heterozygous mutations, c.944G>A, p.Cys315Tyr and c.856C>T, p.Gln286Xaa, in the *TSFM* gene of patients with juvenile-onset Leigh disease, ataxia, neuropathy, and optic atrophy, were reported to lead to EFTU protein degradation and marginally increased mitochondrial protein translation activity (Ahola et al., 2014). Recently, the first case of a patient with a childhood-onset chorea caused by complex heterozygous mutation of *TSFM* (MIM^∗^604723) without basal ganglis lesions was reported (van Riesen et al., 2021).

A homozygous mutation of EF-TS (p.Cys997Thr) was found in both patients with encephalomyopathy and hypertrophic cardiomyopathy. The mutation resulted in mitochondrial translation defects and reduced amounts of assembled complexes I, IV, and V in fibroblasts (Smeitink et al., 2006). SNP genotyping has been used to detect homozygous *TSFM* mutations. The p.Arg312Trp substitution changed arginine to tryptophan, suggesting that mitochondrial translation deficiency is an increasingly serious cause of infant liver failure (Vedrenne et al., 2012). Another homozygous missense mutation was found in the mitochondrial translation elongation factor *TSFM* gene in a patient with slow progressive childhood ataxia and hypertrophic cardiomyopathy (Ahola et al., 2014; Emperador et al., 2016). Whole-exome sequencing (WES) was used to identify mutations in the *TSFM* gene leading to p.Gln111ThrfsTer5 and RNA mis-splicing in a patient with rare mitochondrial disorders (Seo et al., 2019). There is evidence that children with hypertrophic or dilated cardiomyopathy who progress slowly due to *TSFM* mutations develop neurological symptoms that include optic-nerve and/or peripheral neuropathy, ataxia, Leigh disease, and others, which are the main manifestations of the disease (Emperador et al., 2016). A next-generation sequencing (NGS)-based multigene panel for mitochondrial dysfunction was used to identify a *TSFM* homozygous variant, c.547G>A, p.Gly183Ser, associated with early onset encephalomyopathy with sensorineural hearing loss and peculiar neuroimaging features. This result showed that EF-TS-mediated mitochondrial protein translation is valuable for studies of mitochondrial diseases in children with neurological and cardiac involvement (Scala et al., 2019). A patient with a novel *TSFM* mutation has been reported to have an adult-onset complex generalized hyperactivity disorder (Traschutz et al., 2019). The collective findings indicate that *TSFM* mutations are the cause of autosomal recessive mitochondrial cardiomyopathy, encephalopathy with optic and/or peripheral neuropathy, ataxia, and Leigh syndrome.

### mtEF-G1

The human genes *GFM1* and *GFM2* encode mtEF-G1 and EF-G2mt, respectively. Both are highly conserved homologs of bacterial translation elongation factor G (EF-G). The mtEF-G1 elongation factor displays mitoribosome translocation activity. The EF-G2mt factor disassembles the mitoribosome at the end of translation to allow a subsequent protein-synthesis cycle (Glasgow et al., 2017).

The sequence homology between the functional human mtEF-G1 and its bacterial counterpart is ∼45%. The main difference is that there is a mito-specific extension of 11 amino acids at the former’s C-terminus (Bhargava et al., 2004). It was shown that the CTE in mtEF-G1 is directly involved in the translocation of the mt-tRNA receptor arm at the A site. The complex of human 55S mitochondrial ribosome and human mtEF-G1 has three different conformational states including an intermediate state and post-translocation state (Koripella et al., 2020). mtEF-G1 is a five-domain GTPase that catalyzes the transfer of peptide-tRNA from the A site of the ribosome receptor to the P site after the formation of peptide bonds, while removing the deacylated tRNA, promoting mRNA translocation, and exposing the next codon (Brito et al., 2015). mtEF-G1, as a translational GTPase, uses the energy of GTP hydrolysis to facilitate the rearrangement of the pre-translocation ribosomes and tRNA movement, so as to accelerate the process of translocation (Chen et al., 2013). Cryo-EM structures provide insights into the structures of elongation complexes from mammalian mitochondria at two different steps of the tRNA translocation reaction using *in vitro* reconstitution systems. Results have shown that mtEF-G1 not only controls the conformational changes in SSU to promote the movement of tRNA, but also causes the large rearrangement of the GTPase-associated center of mitochondrial LSU. mtEF-G1 binding leads to GAC closure, which stabilizes mtEF-G1 from a weakly to a tightly bound state with translocation ability (Kummer and Ban, 2020).

A study described two new mutations in *GFM1*, which resulted in decreased levels of mtEF-G1, abrogated assembly of mitochondrial complexes III and V, and decreased activity of mitochondrial complexes I and IV. These changes manifested as OXPHOS defects with complex clinical manifestations (Brito et al., 2015). However, the residual steady-state level of mtEF-G1 protein found in the heart and skeletal muscle was higher than that in the liver and fibroblasts, which reduced the sensitivity of these tissues and accentuated the nerve and liver involvement. The difference in residual protein levels among cells may be due to differing regulatory and compensatory responses of the mitochondrial translation system in different tissues (Brito et al., 2015). A tissue-specific effect of novel *GFM1* mutations has been described in three patients by other investigators. In these patients, the respiratory chain enzyme activity of muscle and fibroblasts decreased slightly, while the liver function was seriously deficient (Ravn et al., 2015). A *GFM1* mutation was found in two siblings with serious defects in mitochondrial translation. This mutation is located in a conserved residue in the GTP binding domain of mtEF-G1 (Coenen et al., 2004). A case report showed a *GFM1* mutation in a patient affected by severe, rapidly progressive mitochondrial encephalopathy. This mutation results in a p.Arg250Trp substitution in the mtEF-G1 G′ subdomain and may block ribosome-dependent GTP hydrolysis (Smits et al., 2011a). Another study showed that patients with *GFM1* mutations were affected by severe lactic acidosis, rapidly progressive and fatal encephalopathy, and early onset Leigh syndrome (Valente et al., 2007). A novel intronic *GFM1* mutation was described. The prognosis of patients with this mutation was poor, with death almost always occurring in infancy, although one child was still alive at 6 years of age (Simon et al., 2017). A comprehensive genomic analysis revealed that *GFM1* is one gene mutation known to cause OXPHOS disease in patients with childhood-onset mitochondrial respiratory chain complex deficiencies (Kohda et al., 2016). Recently, a study reported a family that carries a novel *GFM1* variant, which is associated with a rare fatal mitochondrial disease. A p.Cys1576Thr mutation in exon 13 of *GFM1* resulted in a premature stop codon at amino acid position 526 (Coenen et al., 2004; Su and Wang, 2020). WES revealed a novel composition of two heterozygous mutations of *GFM1* in a Chinese child with epilepsy and mental retardation (You et al., 2020). In recent years, nine unrelated children were found to carry *GFM1* mutation. All of these patients presented with nervous system involvement during the neonatal period, and five of them were diagnosed with West syndrome. mtEF-G1 expression was decreased, mitochondrial translation was impaired, and OXPHOS protein levels were decreased in these patients (Barcia et al., 2020). In conclusion, *GFM1* is one of the known gene mutations causing OXPHOS disease, and its mutations and abnormal expression are closely related to a series of mitochondrial diseases.

### GUF1

Bacterial and organellar translation employ a specific regulatory mechanism that differs from that of eukaryotes, involving the highly conserved EF4 translation elongation factor. In this phase of bacterial protein synthesis, EF4 catalyzes the back-translocation of P- and E-tRNAs to A- and P-tRNAs (Zhang et al., 2016). EF4 was originally termed lepA, because the gene encoding the protein is the first cistron of the bicistronic lep operon leader peptidase (lepB or lep) (March and Inouye, 1985). EF4 is highly conserved among all bacteria and almost all eukaryotes. In bacteria, EF4 catalyzes the translocation of peptidyl-tRNA and deacylated-tRNA in the opposite direction of EF-G catalysis. Therefore, EF4 is a back-translocase that maintains translation fidelity by back-translocating the ribosome under stress conditions (Qin et al., 2006). The *E. coli* lepA translation elongation factor has a mitochondrial homolog, mtEF4. Initial studies on budding yeast identified mtEF4 as an evolutionarily conserved GTPase with unknown function. Accordingly, it was also named GTPase of unknown function 1 (GUF1) (Bauerschmitt et al., 2008). In eukaryotes, the N-termini of GUF1 homologs have a mitochondria-targeting signal localized to the mitochondrial outer membrane and are considered to be mtEF4. Under suboptimal conditions, such as low temperatures and high Mg^2+^ concentrations, GUF1 mutant yeast displayed enhanced mitochondrial protein synthesis. At higher temperatures, the assembly of cytochrome c oxidase was shown to be defective in GUF1-deficient mutants (Bauerschmitt et al., 2008). The observations that mtEF4 ablation can reduce the mitochondrial translation rate and disrupt the assembly of complex IV-containing supercomplexes support the important roles of mtEF4 in mitochondrial translation and adaptation to stressful conditions, suggesting that mtEF4 is a key protein that maintains the fidelity of mitochondrial protein synthesis (Bauerschmitt et al., 2008; Yang et al., 2014). It has been shown that mtEF4 is essential for the quality control of respiratory chain biogenesis. Dysregulation of mitochondrial translation caused by its overexpression may be crucial in the development of human cancers. Different mtEF4 levels induce distinct bioenergetic pathways in cancer cells due to different types of speed-quality imbalances. Specific downregulation of mtEF4 expression in tumor tissue could be a promising new therapy for cancer treatment (Zhu et al., 2018). Other authors used exome sequencing to detect mutations in relatives of patients with isolated West syndrome. A homozygous variant (c.1825G>T, p.Ala609Ser) was identified in *GUF1* in three affected siblings (Alfaiz et al., 2016).

In *Caenorhabditis elegans*, a *GUF1* deletion resulted in delayed growth, and MRC complex assembly defects resulted in mitochondrial dysfunction (Yang et al., 2014). We demonstrated testis-specific dysfunction in OXPHOS by genetic ablation of mtEF4 in mice, leading to male infertility (Gao et al., 2016). Our observations demonstrated crosstalk between the mtEF4-dependent quality control in mitochondria and cytoplasmic mammalian target of rapamycin (mTOR) signaling. We showed that with mtEF4 ablation, the main feedback signal from the somatic cytoplasm was mTOR-upregulated and was accompanied by increased cytoplasmic translation, indicating that mTOR is a critical downstream effector compensating for mitochondrial translation deficiency (Gao et al., 2016). We concluded that cytoplasmic translation regulated by mTOR and mitochondrial translation involving mtEF4 have a bidirectional causal relationship. The essential function of mtEF4 provides a plausible explanation for its high degree of evolutionary conservation throughout eukaryotes. Our findings suggest a disease mechanism involving developmental decoupling of crosstalk, such as during spermatogenesis in the testis or pathological conditions in other tissues. Thus, mtEF4 could be a biomarker for human diseases and a drug target for male contraception (Gao et al., 2016).

## Mitochondrial Translation Termination/Ribosome Recycling

### Mitochondrial Translation Termination

Once the translation complex reaches the stop codon, the finished protein must be separated from the final tRNA, ribosome, and its homologous mRNA. In human mitochondria, UGA is not a stop codon, but a tryptophan codon. It is striking that AGA and AGG are not arginine codons, but terminating codons, only at the very end of open reading frames (ORFs) of the mitochondrial transcripts *MT-CO1* (also named *COX1*) and *MTND6* (also named *ND6*), respectively. This results in the possibility of a −1 frameshifting mechanism at the termination stage (Richter et al., 2010a). The remaining 11 mitochondrial ORFs are terminated by either the standard stop codon UAA or UAG. Several release factors are required during this termination. The proteins responsible for these functions are termed release factors (RFs). They recognize mRNA stop codons on the ribosome and control termination of protein synthesis (Soleimanpour-Lichaei et al., 2007). When a termination codon appears at the A site, RFs bind to the ribosome and promote ribosomal PTC-dependent hydrolysis of the ester bond of the peptide-based tRNA binding to the P site (Kisselev et al., 2003). Human mitochondria harbor four different members of the class 1 RF family: MTRF1L, MTRF1, MTRFR (previously called C12orf65), and MRPL58 (previously called ICT1) (Akabane et al., 2014). Mammalian MTRF1L is similar to the bacterial RF sequence. The absence of MTRF1L in human cells leads to growth deficiency (Soleimanpour-Lichaei et al., 2007). Changes in MTRF1L levels related to the mitochondrial inner membrane affect assembly of the respiratory complex and ROS production. MTRF1L is responsible for decoding the UAA/UAG termination codon (Kaji et al., 2001), which enables a single MTRF1L to terminate the translation of all 13 mtDNA-encoded peptides. This is sufficient to release all new human mitochondrial gene products from mitoribosomes (Rao and Varshney, 2001). Further analysis of other mitochondrial factors related to mitochondrial translation termination, such as RRFs, will help us to understand the process of mitochondrial translation termination in mammals and the role of MTRF1L (Nozaki et al., 2008). Desai et al. (2020) described the structure of MTRFR and mitochondrial LSU in rescuing the mitoribosome stalled state by analysis of cryo-EM structures of elongating mitoribosomes and revealed that MTRFR ejects the nascent chain (Desai et al., 2020).

The MRPL58 protein is a component of the human mitoribosome. It has codon-independent peptidyl-tRNA hydrolysis activity through its conserved Gly-Gly-Gln (GGQ) motif. Its function is crucial for hydrolysis of peptidyl-tRNAs that have been prematurely terminated in mitoribosomes and for cell viability (Richter et al., 2010b). *MRPL58* gene knockout results in apoptosis, decreased mitochondrial membrane potential and mass, and decreased cytochrome c oxidase activity (Handa et al., 2010). The MTRFR protein is similar to MRPL58 and plays a similar role in rescuing stalled mitoribosomes. Its knockdown can increase ROS production and apoptosis, thus inhibiting cell proliferation. Compared with control cells, the mitochondrial membrane potential and mass of MTRFR knockout cells change considerably. These results indicate that the function of MTRFR is crucial for cell viability and mitochondrial function (Kogure et al., 2012). Some human diseases are caused by a disorder of translation termination in mitochondria (Temperley et al., 2003). MTRFR participates in the process of mitochondrial translation and is related to multiple phenotypes, including early onset optic atrophy, progressive encephalomyopathy, peripheral neuropathy, and spastic paraparesis (Fang et al., 2017a). Loss of *MTRFR* gene function causes mitochondrial translation defects, leading to encephalomyopathy (Temperley et al., 2003). Another study identified a 2-base deletion in *MTRFR* in a Japanese woman with mitochondrial dysfunction in choroid plexus cell bodies (Nishihara et al., 2017). Other groups identified a novel protein-truncating mutation in the *MTRFR* gene in a family with neuropathy and optic atrophy. Cells from these individuals exhibited mitochondrial defects, including reduced mitochondrial respiration complex activity and stability, decreased mitochondrial respiration rate, and decreased mitochondrial membrane potential (Tucci et al., 2014). Another group reported that a compound heterozygous mutation of *MTRFR* caused distal motor neuropathy and optic atrophy in a Chinese patient (Fang et al., 2017b). Siblings diagnosed with combined OXPHOS deficiency type 7 (COXPD7) had *MTRFR* compound heterozygous mutations. They displayed optic atrophy, mild developmental delays, and bilateral brainstem symmetry (Heidary et al., 2014). Homozygosity mapping was used to identify mutations in *MTRFR* in two patients who developed Leigh syndrome, optic atrophy, and ophthalmoplegia. Analysis of mitochondrial translation in fibroblasts from these patients revealed decreased mitochondrial translation, with considerable decreases in complexes I, IV, and V, and with a slight decrease in complex III (Antonicka et al., 2010). Finally, the analyses of two affected siblings with mild intellectual disability, spastic paraplegia, and strabismus revealed a homozygous premature stop mutation at codon 139 of *MTRFR* using homozygosity mapping and exome sequence analysis (Buchert et al., 2013). A homozygous nonsense mutation of *MTRFR* (NM_001143905:c.346delG, p.Val116^∗^) was described in a pair of female twins diagnosed with Leigh syndrome (Imagawa et al., 2016). Other groups described four patients with the classical Behr syndrome phenotype who had homozygous nonsense mutations in the *MTRFR* gene (Pyle et al., 2014). A novel *MTRFR* mutation was identified in seven affected individuals from two closely related families by whole-genome homozygosity mapping and exome sequencing. Disruption of the GGQ domain in the first coding exon led to a more severe phenotype (Spiegel et al., 2014). In one case, a new pathologic variant of the *MTRFR* gene was identified. The mutant protein lacked the GGQ domain (Perrone et al., 2020). The homozygous pathologic variant of *MTRFR* displayed a damaged mitochondrial OXPHOS system. The findings indicated that loss-of-function variants are more likely to lead to disease, while variations affecting the GGQ domain are associated with more severe phenotypes (Perrone et al., 2020). The results of these two cases are consistent, indicating that the GGQ domain of MTRFR is crucial for its phenotype, and its deletion causes a more severe phenotype.

### Mitochondrial Translation Recycling

After the termination of protein synthesis, the mRNA and deacylated-tRNA in the peptide/exit (P/E) state remain associated with the ribosome to form the post-termination complex (PoTC) (Kaji et al., 2001). To start a new round of protein synthesis, the ligands binding to ribosomes must be removed from the PoTC and the ribosomes must separate into their two subunits. In this process, the RRF and EF-G2mt cooperate to disassemble the PoTC (Rao and Varshney, 2001), with the EF-G2mt transferred to the A site together with RRF to catalyze the release of mRNAs, deacylated-tRNA, and ribosome subunits, which are necessary for ribosome recycling (Hansen et al., 2000). The binding of EF-G2mt⋅GTP to the RRF-PoTC results in the disassembly of the 55S ribosome into two subunits during GTP hydrolysis (Peske et al., 2005). The cryo-EM structure of the human 55S mitoribosome-RRF complex revealed that the mito-specific NTE of RRF has α-helix and loop structures. These structures produce a functional key region that interacts with mitochondrial ribosomes. The structure revealed the presence of a tRNA at the P/E position and the rotation of small mitochondrial ribosomal subunits upon RRF binding. The research also revealed the interaction between P/E tRNA and mL64. These findings help to understand the unique features of the mitochondrial ribosome cycle (Koripella et al., 2019b). Furthermore, a recent paper mentioned the role of GTP binding protein 6 (GTPBP6), a homolog of the bacterial ribosome recycling factor HflX, in the division of ribosomal subunits, especially under stress conditions. This study showed that GTPBP6 plays a dual role in the ribosome cycle and biogenesis. On the one hand, it is conducive to the dissociation of ribosomes; on the other hand, it promotes the assembly of mitochondrial ribosomes. These findings contribute to our understanding of the assembly of large ribosomal subunits and the mitochondrial ribosomal recycling pathway (Lavdovskaia et al., 2020).

The EF-G2mt [also designated RRF2mt and GFM2 (Takeuchi et al., 2010)] protein mediates ribosomal recycling together with human RRF, but lacks translocation activity. The functional specificity of EF-G2mt involves domains III and IV. Therefore, EF-G2mt represents a class of guanosine triphosphate hydrolases (GTPases) involved in ribosome recycling. It cooperates with RRF and mediates ribosome dissociation during ribosome recycling, which is essential for the recycling stage.

The MRRF protein (also called RRF) was previously named mtRRF-1 (Takeuchi et al., 2010). It is a GTP-binding protein. Its binding can stabilize the rotational conformational state of mitoribosomes and has multiple weakened specific subunit bridges, preparing the complex for the dissociation of the EF-G2mt-binding subunits (Zhang and Spremulli, 1998). Deletion of MRRF in yeast does not decrease mitochondrial protein synthesis or stability of mtDNA. Thus, MRRF is involved in the coordination between yeast mitochondrial translation and OXPHOS assembly (Ostojic et al., 2016). Only the combination of EF-G2mt and MRRF can rescue the temperature-sensitive characteristics of *E. coli* RRF (Qin et al., 2006).

The MRRF protein is essential for the survival of human cell lines. Depletion of MRRF in human cell lines is fatal, which initially leads to severe mitochondrial abnormalities, mitoribosome aggregation, increased mitochondrial superoxide production, and eventual loss of the OXPHOS complex. Hence, MRRF loss results in decreased growth rate and cell death, leading to a variety of mitochondrial dysfunctions and diseases (Rorbach et al., 2008). Two biomarkers, RRF and ribosomal protein S18 (RPS18), distinguish early PD from normal control samples and are thus considered high-confidence biomarkers of distinct protein autoantibodies for early PD (Wu et al., 2020). These findings could help establish a timely and accurate method for the diagnosis of early PD (Wu et al., 2020). Mutations in *GFM2* have been found in patients with Leigh syndrome. *GFM2* mutations (c.206 + 4A>G and c.2029-1G>A) were found in both siblings, resulting in abnormal splicing of the premature stop codon (p.Gly50Glufs^∗^4 and p.Ala677Leufs^∗^2, respectively). Thus, the *GFM2* mutation may be the cause of Leigh syndrome with multiple congenital arthritis phenotypes (Fukumura et al., 2015). WES was used to identify compound heterozygous (c.569G>A, p.Arg190Gln; c.636delA, p.Glu213Argfs^∗^3) and homozygous (c.275A>C, p.Tyr92Ser) recessive variants of *GFM2* in patients presenting in early childhood with global developmental delay, elevated cerebrospinal fluid levels of lactate, and abnormalities on cranial magnetic resonance imaging (Zhang et al., 2016). Further research also identified these recessive *GFM2* variants in two unrelated patients with early-onset neurological presentations of mitochondrial disease (Glasgow et al., 2017).

## Mitoribosomes and Related Disease

### Mammalian Mitoribosomes and Mitochondrial Ribosome Assembly

Unlike 70S ribosomes in prokaryotes and 80S ribosomes in the cytoplasm of eukaryotes, human (mammalian) mitochondria contain 55S ribosomes (O’Brien, 2003). The mitochondrial genome encodes both 12S rRNA and 16S rRNA, but all MRPs are encoded by the nuclear genome. The rRNAs have catalytic function, while ribosomal proteins have not only structural but also biological function in the process of translation. The MRPs are imported into mitochondria and assembled with the rRNAs transcribed by the mitochondria to form ribosomes responsible for translating mRNAs of 13 essential proteins in the OXPHOS system (O’Brien, 2003). The bovine 55S mitochondrial ribosome with a molecular weight of 2.71 MDa consists of two subunits of different sizes: a small subunit (28S) and large subunit (39S) (O’Brien, 1971). Compared with bacterial 30S consisting of 16S rRNA (1,542 nucleotides) and 21 proteins (S1–S21), the 28S SSU contains a 12S rRNA (950 nucleotides) and 29 proteins (Suzuki et al., 2001). Compared with bacterial 50S composed of two rRNA molecules (5S, 120 nucleotides; 23S, 2904 nucleotides) and 33 proteins (L1–L36), the 39S LSU of mitoribosomes contains a 16S rRNA (1560 nucleotides) and 48 proteins (Koc et al., 2001). Therefore, compared with bacterial ribosomes (33% protein and 67% RNA), the ratio of MRP to RNA is completely reversed, with 69% protein and 31% RNA. Of the 77 mitoribosomal component proteins, almost half are mitoribosome-specific and the remainder are bacterial protein homologs. Sharma et al. (2003) first analyzed the 3-dimensional cryo-EM map of the bovine mitochondrial 55S ribosome with a resolution of 13.5 Å. It was found that many proteins occupied a new position in ribosomes. Mitochondrial ribosomes have intersubunit bridges composed of proteins and have a gate-like structure at the mRNA entrance, which may be involved in the recruitment of unique mitochondrial mRNAs (Sharma et al., 2003). Subsequently, in 2009, the mitochondrial ribosomes of *Leishmania tarentolae* were reconstructed with a resolution of 14.1 Å. Greber et al. (2015) published the complete structure of the porcine 28S mitoribosome SSU and the reconstruction model of the 55S mitoribosome complexes with mRNA and tRNA. This structure revealed that the interaction between subunits in mitochondria is not as extensive as that in bacteria, and many bridges are formed by mitochondria-specific RNA and protein components. Reduced peripheral contacts may result in increased conformational flexibility of mitoribosomal subunits, including relative tilt between subunits (Greber et al., 2015). Subsequent studies confirmed the previous structure discovery that the large reduction in ribosomal RNA led to topological changes in some function-related regions in mammalian mitoribosomal structures, including the tRNA binding sites and nascent polypeptide-exit tunnels (Kaushal et al., 2015).

Despite the high-resolution mitoribosomal structures, the problem of how these macromolecular structures are assembled remains. Ribosome assembly involves the coordinated processing and modification of the time-related relationship between rRNAs and ribosomal proteins (De Silva et al., 2015). Many ribosome assembly factors act as macromolecular machines to improve efficiency and provide higher levels of control over mitochondrial translation. Mitochondrial assembly factors include GTPases, helicases, pseudouridine synthases, methyltransferases, endonucleases, and factors without known enzyme activity (Lopez Sanchez et al., 2021). To date, many mitochondrial assembly factors are RNA-binding proteins related to 12S and 16S rRNA, which have molecular chaperone activity and help them fold correctly (Lopez Sanchez et al., 2021). Studies have shown that mitoribosome assembly factors play a very important role in the process of mitochondrial translation, and are involved in diseases caused by mitochondrial translation disorders. Next-generation WES helps to identify pathogenic mutations in nuclear genes of different components of the mitochondrial protein translation machinery, including mitoribosome biogenesis and assembly. Pathogenic mutations have been identified in the RNA components of the mitoribosome (12S, 16S, and CP-tRNA^Val^), MRPs, and mitoribosome assembly factors. These mutations are associated with a wide range of clinical features, can be present at all stages of life, and are associated with variable tissue specificity (Lopez Sanchez et al., 2021).

### MRPs Mutations

In 2004, it was reported for the first time that the nonsense mutation of mitochondrial SSU protein S16 (*MRPS16*) gene significantly reduced the transcription level of 12S rRNA, resulting in mitochondrial protein translation defects. This report found a case of neonatal lactic acidosis with agenesis of corpus callosum, dysmorphism, and lethality. The activities of complexes I and IV in the patient’s muscle and liver were significantly decreased, accompanied by extensive mitochondrial translation defects. Analysis of the patient showed a homozygous C-to-T substitution at nucleotide 331 of the *MRPS16* cDNA (Miller et al., 2004). Similar results showed that in *Drosophila*, a missense mutation in mitochondrial ribosomal protein S12 prevented ribosomal proteins from assembling into active ribosomes, resulting in a significant reduction in 12S rRNA transcripts (Toivonen et al., 2001). Another mitochondrial ribosomal SSU protein mutation occurs in *MRPS22*. That report identified *MRPS22* gene mutations in patients with antenatal skin edema, hypotonia, cardiomyopathy, and tubulopathy born to the same set of consanguineous parents. Transfection with wild-type *MRPS22* cDNA could increase 12S rRNA content and normalize enzyme activity (Saada et al., 2007). Another study also reported that a patient with Cornelia de Lange-like dysmorphic features, brain abnormalities, and hypertrophic cardiomyopathy had a mutation in *MRPS22*. This study found that a mutation at a conserved site in the *MRPS22* gene resulted in a p.Leu215Pro substitution, which seriously damaged the mitochondrial protein translation in fibroblasts and caused defects in OXPHOS complexes I, III, and IV. The amount and activity of OXPHOS complex IV and the transcription level of 12S rRNA could be restored to normal levels by transfection to increase the expression of MRPS22 in fibroblasts (Smits et al., 2011b). Researchers investigated phenotypes of mice carrying a homozygous mutation in mitochondrial ribosomal protein of small subunit 34 (*MRPS34*) and found that the mutant mice developed cardiac hypertrophy and liver steatosis with age. MRPS34 is one of 15 mammalian mitochondria-specific MRPs, which has not been found in the ancestors of bacterial ribosomes (Greber et al., 2015). Further studies have shown that MRPS34 is required for mitochondrial translation, stability of small ribosomal subunits, and its association with the large subunit. The *MRPS34* mutation caused an obvious decrease in this protein, resulting in reduced levels of mitochondrial proteins and complexes, which led to decreased oxygen consumption and respiratory complex activity (Richman et al., 2015). The research group then identified the *MRPS34* autosomal-recessive mutations in six individuals in four families with OXPHOS deficiency and Leigh syndrome or Leigh-like disease. Further investigation showed that these mutations caused reduced mitochondrial translation and combined OXPHOS deficiency by destabilizing the small mitochondrial ribosome subunit (Lake et al., 2018).

Galmiche et al. (2011) first identified mutations of *MRPL3*, the first large ribosomal subunit protein, through WES of individuals with multiple respiratory chain defects. The compound heterozygotes with a missense *MRPL3* mutation (p.Pro317Arg) and a large-scale deletion were shown to have altered ribosome assembly and mitochondrial translation defects in skin fibroblasts, resulting in abnormal assembly of several respiratory chain complexes (RCC). The investigators also showed that these *MRPL3* mutations were the cause of severe hypertrophic cardiomyopathy in four siblings born to non-consanguineous parents (Galmiche et al., 2011). NGS exome sequencing of two siblings with recessive hypertrophic cardiomyopathy uncovered a homozygous mutation (p.Leu156Arg) in the *MRPL44* gene. MRPL44 is one of 20 mitochondrial ribosomal LSU proteins without a bacterial homolog and is reported to be located near the tunnel exit of the mitochondrial ribosome in yeast. Missense mutations in the *MRPL44* gene affect protein stability, resulting in a severe decline in MRPL44 levels in the heart, skeletal muscle, and fibroblasts of patients. In patient fibroblasts, the reduction in MRPL44 has no effect on *de novo* mitochondrial translation, but seriously affects the assembly of large ribosomal subunits and the stability of 16S rRNA, resulting in the lack of complex IV. These results indicate that MRPL44 directly affects the assembly and stability of nascent mitochondrial polypeptides such as COX1 and likely interacts with chaperones or assembly factors. These studies suggest that some mitochondrial ribosomal subunit defects can produce tissue-specific phenotypes such as cardiomyopathy (Carroll et al., 2013). This conclusion was further confirmed by another study, which found that the heart muscle is particularly vulnerable to metabolic defects. This study expands the clinical spectrum of mitochondrial diseases associated with MRPL44 and indicates that the defect also leads to slowly progressive multisystem diseases, including those of skeletal muscle, liver, kidney, and the central nervous system. Therefore, *MRPL44* mutations are more likely to occur in patients with slowly progressing mitochondrial multisystem diseases, especially in patients with cardiomyopathy (Distelmaier et al., 2015). In addition, one research group sequenced the mitochondrial ribosomal protein L12 (*MRPL12*) gene in a patient who was born to consanguineous parents and presented with growth retardation and neurological deterioration; they identified a c.542C>T missense mutation in exon 5, which converted a highly conserved alanine to valine (p.Ala181Val). This mutation led to decreased MRPL12 protein levels, which affected the assembly of large ribosomal subunits, caused overall mitochondrial translation defects, and significantly reduced the synthesis of COX1, COX2, and COX3 subunits. Studies in eubacteria have shown that eubacterial L7/L12, which are MRPL12 homologs, play an important role in protein synthesis by interacting with translation factors and regulating the speed and accuracy of protein synthesis (Pettersson and Kurland, 1980; Dey et al., 1995; Helgstrand et al., 2007). Modeling of MRPL12 showed that the p.Ala181Val change may alter the binding with elongation factor, thus reducing the affinity of mutant MRPL12 for the ribosome (Serre et al., 2013).

## mt-tRNA (Mt-T) Mutations and Disease

### mt-tRNA (MT-T) Overview

Among the 37 genes encoded by mtDNA, 13 encode electron transfer chain components, 2 encode mt-rRNAs, and the remaining 22 encode mt-tRNA (MT-T) genes. The *MT-T* gene has a unique secondary structure and forms endonuclease sites (Ojala et al., 1981; Taanman, 1999). After transcription, 14 cytosine-rich “light” mt-tRNAs and 8 guanine-rich “heavy” mt-tRNAs are released into the matrix (Anderson et al., 1981; Larsson and Clayton, 1995; Taanman, 1999). The secondary structure of tRNAs typically consists of a cloverleaf-shaped base-pairing pattern containing four domains: the acceptor stem, D-stem/loop, TψC-stem/loop, and anticodon stem/loop. Compared with cytoplasmic or bacterial tRNAs, the intrinsic thermodynamic stability of mt-tRNAs is decreased, mainly due to decreased GC content and increased non-Watson–Crick base pair frequency in the stem region. Thermodynamic instability may lead to inactivation of mt-tRNAs due to pathologic mutations; a single base substitution is more likely to destroy these weaker structures than structures with stronger contact sets (Wittenhagen and Kelley, 2003). There are more than 300 pathological mutations in the region of mtDNA encoding the *MT-T* gene. Although these mutations are widely distributed in the *MT-T* gene, three tRNAs, namely mt-tRNA^Ile^ (MT-TI), mt-tRNA^Leu^ (UUR) (MT-TL1) and mt-tRNA^Lys^ (MT-TK), contain almost 50% of the known pathologic mutations (Wittenhagen and Kelley, 2003).

### mt-tRNA (MT-T) Mutations

*MT-T* gene mutations are associated with many clinical characteristics, many of which also occur in mitochondrial diseases (Taylor and Turnbull, 2005). In general, there is little correlation between *MT-T* gene mutations and clinical manifestations. Mutations at different sites of the same *MT-T* gene may lead to completely different clinical symptoms, and the same point mutation may also lead to several different clinical phenotypes. The MITOMAP and Mamit-tRNA databases show the clinical variability of *MT-T* mutations, and mutations in different *MT-T* genes can lead to the same clinical presentation. For example, MELAS, chronic progressive external ophthalmoplegia (CPEO), and maternally inherited diabetes and deafness (MIDD) are reported clinical syndromes of m.3243A>G mutation within *MT-TL1*, which are also closely related to other *MT-T* gene mutations. Studies have identified many different possible effects of mutations, including interference of 3′ terminal maturation, prevention of aminoacylation, disruption of transcription factor binding, and codon recognition (Florentz et al., 2003). However, the basic molecular mechanism of mutation leading to disease is not well understood (Zifa et al., 2007). The only common feature of *MT-T* mutation is the loss of stability of MT-T. Among all the mutants, m.8344A>G and m.3243A>G are the two most common heteroplasmic *MT-T* genetic variants. Because pathologic changes usually occur in Watson–Crick pairs, which are structurally important in stems, it is unexpected that both mutations occur within loop structures (Yarham et al., 2010).

m.8344A>G, located in the T loop of mt-tRNA^Lys^, was the first pathologic heteroplasmic mutation of the *MT-T* gene to be identified (Shoffner et al., 1990). m.8344A>G is the sole cause of mitochondrial protein synthesis defects (Chomyn et al., 1991). Mutant transmitochondrial cybrids showed 10-fold reduced oxygen consumption, decreased cytochrome c oxidase activity, and respiratory defects caused by impaired protein synthesis (Chomyn et al., 1991; Masucci et al., 1995). Heteroplasmic mutation of the *MT-T* gene is closely related to the myoclonic epilepsy with ragged-red fiber (MERRF) phenotype. However, the mechanism of m.8344A>G mutation causing this phenotype has not been determined. One report suggested that this mutation causes a decrease in mt-tRNA^Lys^ steady-state levels (Masucci et al., 1995). It was also reported that m.8344A>G causes a decrease in aminoacylation, which may be the primary cause of protein synthesis defects (Enriquez et al., 1995). Some studies have shown that m.8344A>G causes the post-transcriptional taurine modification defect of wobble position uridine in mt-tRNA^Lys^, which is also a reason for mitochondrial protein synthesis defects (Enriquez et al., 1995; Yasukawa et al., 2000a). Therefore, we suggest that the m.8344A>G mutation leads to the MERRF phenotype mainly by preventing the post-transcriptional taurine modification of wobble position uridine. m.8344A>G weakened the codon-anticodon interaction in mt-tRNA^Lys^, stalled translation, and reduced protein synthesis. Furthermore, ribosomal shifting may then occur, leading to premature translation termination and resulting in abnormal translation products. Although m.8344A>G is the most common mutation associated with MERRF (∼80% of cases) (Ozawa et al., 1997), other mutations such as m.8356T>C have also been reported (Masucci et al., 1995).

Mitochondrial disease MELAS syndrome is usually associated with many *MT-T* gene point mutations. Approximately 80% of MELAS syndrome patients had m.3243A>G mutations in the *MT-TL1* gene (Morgan-Hughes et al., 1995). The m.3243A>G mutation occurs in the mtDNA binding site of MTERF1, which leads to a decrease in MTERF1 affinity, resulting in mitochondrial protein synthesis defects and respiratory disorders (Chomyn et al., 1992; King et al., 1992). As was previously observed with m.8344A>G, m.3243A>G mutation caused mt-tRNA^Leu^(UUR) molecules to lack wobble position uridine modification (Yasukawa et al., 2000b, 2002). Because wobble uridine is considered to play an important role in stability, this mutation leads to protein synthesis defects, resulting in the MELAS phenotype (Yasukawa et al., 2002; Sasarman et al., 2008). Interestingly, the m.3243A>G. mutation enhanced the dimerization of mt-tRNA^Leu^ (UUR), resulting in a 10-fold reduction of aminoacylation in dimers (Wittenhagen and Kelley, 2002). Hence, decreased aminoacylation and post-transcriptional modification defects are crucial to causing respiratory deficiency and MELAS syndrome (Yasukawa et al., 2000b).

## Mitochondrial Aminoacyl-tRNA Synthetases (mt-aaRss)

Aminoacyl tRNA synthetases (aaRSs) are a group of nuclear-encoded enzymes that conjugate each of the 20 amino acids to their cognate tRNA molecules to ensure correct translation of the genetic code (Sissler et al., 2017; Ognjenovic and Simonovic, 2018). mt-aaRSs are imported into the mitochondrial matrix and supply mt-tRNA conjugates for protein translation. In principle, each amino acid is recognized by its specific aaRS, resulting in 20 aaRS protein synthesis systems per cell. However, in human mitochondria, only 19 aaRSs are present, because Gln-tRNA^Gln^ is synthesized indirectly via misacylated Glu-tRNA^Gln^ through transamidation (Nagao et al., 2009). Recently, mutations in the genes encoding mt-aaRSs have been identified as a new cause of human diseases. They are believed to impair mitochondrial protein synthesis, thereby affecting the OXPHOS system and leading to surprising tissue-specific phenotypes. At present, nine gene mutations encoding mitochondria-specific aaRSs have been reported. Among these mutations, encephalopathy is the most common phenotype, while cardiomyopathy, tubulopathy, myopathy, or sensorineural neuropathy is the next most common phenotype (Konovalova and Tyynismaa, 2013). *DARS2*, *EARS*, and *AARS2* are three typical genes whose mutations lead to rare and well-defined leukodystrophy (LD) syndrome. The mutations occur in mitochondrial aspartyl-tRNA synthetase, mitochondrial glutamate tRNA synthetase, and mitochondrial alanyl-tRNA synthetase, respectively (Fine et al., 2019). Mt-aaRSs as key players in the mitochondrial translation machinery play an important role in cell energy production. In AARS2 ovarian LD, leukoencephalopathy with thalamus and brainstem involvement and high lactate (LTBL), and leukoencephalopathy with brainstem and spinal cord involvement and lactate elevation (LBSL), protein and enzyme activities are reduced to varying degrees, but are not completely lacking. For AARS2 ovario-LD and LTBL, RCC dysfunction occurred, which was not detected in LBSL patient cells (Scheper et al., 2007; Mikhailova et al., 2009; Synofzik et al., 2011; Taskin et al., 2016). In addition, in LTBL, the oxygen consumption rate decreased significantly (Fine et al., 2019). Moreover, *AARS2* was found to be the disease gene for early-onset fatal hypertrophic cardiomyopathy with lactic acidosis (Gotz et al., 2011). The patients died during the perinatal period or within 10 months after birth. Cardiomyopathy was a prominent clinical manifestation, but in addition to the heart, OXPHOS deficiency in the brain and muscle was observed at autopsy. The patients, however, did not show OXPHOS deficiency in fibroblasts or myoblasts. *AARS2* mutations have been identified as homozygous or heterozygous, but to date, all the patients described have a mutation leading to p.Arg592Trp in mitochondrial alanyl-tRNA synthetase (Gotz et al., 2011; Calvo et al., 2012). Mt-aaRSs not only promote the translation of proteins forming the mitochondrial respiratory chain complex, but they also affect cell signaling, transcription, and RNA biological genesis in neurons (Sissler et al., 2017). At present, many studies have reported the non-canonical effects of mt-aaRSs, such as the angiogenic function of rat mitochondrial tryptophanyl-tRNA synthetase (WARS2) and the possible role of mt-aaRS mutations in the integrated stress response (ISR) (Agnew et al., 2018). Complete inhibition of mitochondrial translation in DARS2 knockout mice leads to the accumulation of unassembled nuclear-encoded respiratory chain subunits, resulting in severe protein homeostasis stress and mitochondrial unfolded protein response (UPRmt)-dependent ISR activation. The ISR does not reach steady state, which leads to severe heart disease and decreased survival (Dogan et al., 2014). In addition to mt-aaRS mutations that affect the pathology of the central nervous system, mt-AlaRS, mt-GlyRS, and mt-LysRS mutations are known to cause cardiomyopathies (Hei et al., 2019; Sommerville et al., 2019); mt-TyrRS mutations caused myopathy, lactic acidosis, and sideroblastic anemia (MLASA syndrome) (Shahni et al., 2013); and mt-SerRS mutations caused hyperuricemia, pulmonary hypertension, renal failure in infancy, and alkalosis (HUPRA syndrome) (Rivera et al., 2013). Generally speaking, the characteristics of mt-aaRSs are relatively poorly understood, and future research will provide important knowledge regarding the function of these synthetases. The detection of identified *AARS2* mutations provides some clues for understanding the molecular mechanism of these rare diseases.

## Mitochondrial Translational Activators

Mitochondrial translational activators are nuclear-encoded proteins. A yeast translational activator was proposed in the late 1980s. These activators generally interact with the 5′-untranslated region (UTR) of mitochondrial mRNA. They may also bind to nascent proteins or interact with mitoribosomes. Translation activators are often bound to the membrane, which may limit translation to the region close to the inner membrane (Fox, 2012). It is not clear whether all translational activators exhibit all of these activities. Unfortunately, due to the lack of an *in vitro* translation system, analyzing their activities in the process of translation initiation and elongation is not yet possible. Some protein factors, such as translational activator of cytochrome oxidase I (TACO1), mitochondrial translation regulation assembly intermediate of cytochrome c oxidase (MITRAC), and COX14 (previously called C12orf62), are recruited to bind directly to mitochondrial transcripts and regulate the translation of MT-CO1 (Szklarczyk et al., 2012; Weraarpachai et al., 2012; Richter-Dennerlein et al., 2016). In yeast, the translational activator of COX2 (MT-CO2) is PET111 (Sanchirico et al., 1998), which is necessary for COX2 mRNA translation (Fiori et al., 2005). PET54, PET122, and PET494 are translational activators of COX3 (MT-CO3). They interact with the 5′-UTR of *COX3* mRNA, the small ribosomal subunit protein, and the inner membrane to promote the synthesis of COX3 (Naithani et al., 2003). The mitochondrial translation activators CBS1 and CBS2 interact specifically with the cytochrome b gene (*MT-CYB* or *COB*) mRNA via its 5′-UTR (Mittelmeier and Dieckmann, 1995). Both activators participate in translation initiation and promote the synthesis of MT-CYB by binding to mRNA-ribosome complexes (Rodel, 1986). CBP1 is another protein that binds to the 5′-UTR of *MT-CYB* mRNA, which is necessary for *MT-CYB* mRNA translation (Dieckmann et al., 1984; Islas-Osuna et al., 2002). In addition, CBP3 and CBP6 participate in the translation of MT-CYB (Gruschke et al., 2011).

Most yeast genes involved in the translation of mitochondria-encoded proteins lack mammalian homologs due to the lack of 5′-UTRs in mammalian mitochondrial mRNAs (Fontanesi et al., 2006). To date, TACO1 is the only specific mitochondrial mammalian translation activator. Expression of TACO1 in fibroblasts rescued the COX1 (MT-CO1) synthesis and assembly defects (Weraarpachai et al., 2009). TACO1 is necessary for the efficient translation of COX1. Investigations on TACO1^mut/mut^ mice showed that TACO1 is required for COX1 translation through its specific binding of *MT-CO1* mRNA and association with mitochondrial ribosomes. These mutant mice developed a late-onset syndrome similar to human patients with visual impairment, motor dysfunction, and cardiac hypertrophy (Richman et al., 2016). MITRAC is a complex IV assembly intermediate that regulates mitochondrial translation. MITRAC was found to interact with multiple assembly factors and efficiently translate *COX1* mRNA (Mick et al., 2012). Lrpl30 and leucine rich pentatricopeptide repeat containing (LRPPRC) are translation activators of the human cytochrome c oxidase subunits (Debray et al., 2011). Both have multiple targets, including COX1 and COX3. Mutations result in a deficiency of cytochrome c oxidase, leading to French–Canadian Leigh syndrome (Kohler et al., 2015).

## Regulation of Mitochondrial microRNAs (mitomiRs) in Mitochondrial Translation and Their Role in Disease

MicroRNAs (miRNAs) are single-stranded non-coding RNAs that are 18–23 nucleotides in length. They can translocate into the mitochondria and regulate mitochondrial translation (Bandiera et al., 2013). MitomiRs regulate mitochondrial gene expression and function under physiological and pathological conditions (Paramasivam and Vijayashree Priyadharsini, 2020). Several mitomiRs may be derived from mitochondrial genomic mRNA. MitomiRs post-translationally regulate gene expression in the mitochondria (Das et al., 2012; Fan et al., 2019). Most importantly, differentially expressed mitomiRs were observed in heart failure (Pinti et al., 2017; Wang X. et al., 2017). However, the translocation mechanism of nuclear-encoded miRNAs into mitochondria is not clear. MitomiR transport is one of the most controversial fields in mitochondrial research. Researchers have questioned the transport of RNA into mitochondria. In recent years, many miRNA processing proteins, including Ago, Dicer and RISC, have been found in mitochondria (Chen et al., 2010; Wang et al., 2015). Knowledge of the mechanism of miRNA transport to the mitochondrial matrix may provide important insights into the pathophysiology of disease and may become a new target for therapeutic intervention.

MicroRNAs miR-1, miR-210, and miR-338 can enhance mitochondrial translation and regulate mitochondrial proteomics and mitochondrial bioenergetics in myocytes (Aschrafi et al., 2008; Colleoni et al., 2013; Zhang et al., 2014; Srinivasan and Das, 2015). In mitochondria, miR-1 unexpectedly stimulates, rather than inhibits, translation of mitochondrial genome-encoded transcripts. The observed positive role of mitomiRs in mitochondrial translation suggests that miR-1 regulates the myogenic program by mediating mitochondrial translational activation and inhibiting cytoplasmic translation. Thus, mitomiRs play an important role in the crosstalk between mitochondrial and cytoplasmic translation. However, another study showed that hypoxia induced the expression of miR-210 in fibroblasts, while the expression of its downstream targets, iron-sulfur cluster assembly enzyme (ISCU) and the COX10 cytochrome c oxidase assembly protein, decreased. Moreover, miR-210 inhibited protein synthesis, thus reducing the level of the electron transport system (ETS) complex protein (Colleoni et al., 2013). MicroRNA-181c (miR-181c) inhibits the translation of COX1, resulting in the remodeling of complex IV and enhancement of mitochondrial function in ventricular myocytes. It regulates the mitochondrial genome and bioenergy and may potentially regulate heart failure *in vivo* (Das et al., 2012, 2014). The microRNA miR-762 translocates into mitochondria and is upregulated during hypoxia/reoxygenation in cardiomyocytes. Therefore, miR-762 can directly reduce MT-ND2 translation, mitochondrial complex I enzyme activity, and ATP levels, and increase ROS levels and cardiomyocyte apoptosis (Yan et al., 2019). It can also be translocated to the mitochondria to inhibit the downregulation of MT-CYB. Overexpression of miR-92a enhances mitochondrial translation and reduces ROS production and lipid deposition, thus improving diabetic cardiomyopathy (Li et al., 2019). Another study reported significant upregulation of miR-21 in spontaneously hypertensive rats. Computational prediction and biochemical analyses revealed that miR-21 directly targets MT-CYB and positively regulates its mitochondrial translation. miR-21 also reduces blood pressure and myocardial hypertrophy in spontaneously hypertensive rats by upregulating the mitochondrial translation level of MT-CYB (Li et al., 2016). Therefore, some mitomiRs promote mitochondrial translation, while others inhibit it. The mechanism of mitomiRs regulating mitochondrial translation needs further study.

## Crosstalk Between Mitochondrial and Cytoplasmic Protein Synthesis

**FIGURE 2:**
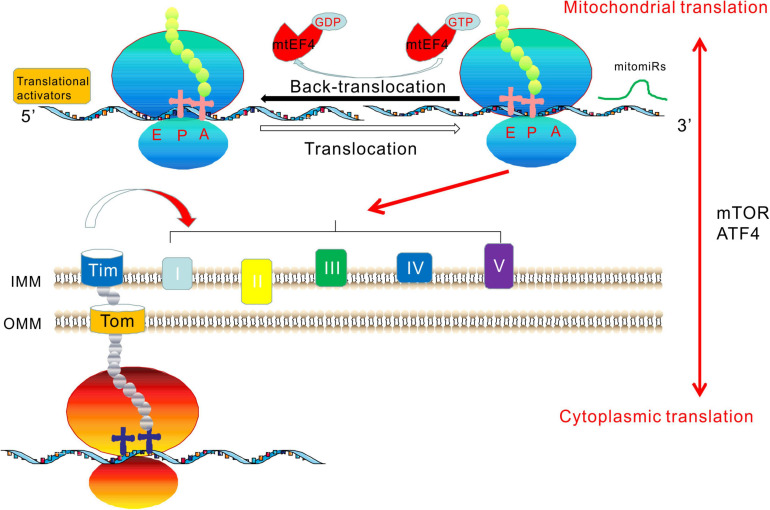
Crosstalk between mitochondrial and cytoplasmic translation. mtEF4, translation activators, and mitomiRs are important regulators that directly affect mitochondrial translation. The components of mitochondrial complexes I, II, III, IV, and V are produced by both mitochondrial and cytoplasmic translation, requiring coordination between the two translation systems. mtEF4 has a back-translocation effect and catalyzes the back-translocation of the P- and E-tRNAs to A- and P-tRNAs. Mitochondrial translation activator promotes mRNA-specific translation initiation. MitomiRs function at the 3′ end of mRNA. mTOR and ATF4 play an important role in the crosstalk between mitochondrial and cytoplasmic translation.

OXPHOS subunits are encoded by both the nuclear and mitochondrial genomes. However, the co-regulation of OXPHOS subunit genes remains poorly understood. This cooperative translation program provides one-way control through complex and dynamic cytoplasmic translation control. Therefore, the nuclear genome precisely guides the coordination of mitochondrial and cytoplasmic translation to coordinate the synchronous synthesis of the OXPHOS complex (Tang et al., 2020). Recently, Guo et al. (2020) identified the molecular pathway of the mitochondrial stress signal relayed to the cytoplasm. In mammalian cells, mitochondrial dysfunction caused by mitochondrial translation disorders leads to an ISR. This ISR is mediated through eIF2α phosphorylation. Phosphorylation of eIF2α decreases overall protein synthesis, but increases the translation of only ATF4, the master transcriptional regulator of ISR. Mitochondrial stress stimulates a mitochondrial stress-activated protease (OMA1)-dependent cleavage of DAP3 binding cell death enhancer 1 (DELE1), a protein found to be associated with the inner mitochondrial membrane. Hence, DELE1 accumulates in the cytosol and interacts with eukaryotic translation initiation factor 2 alpha kinase 1 (HRI or EIF2AK1), an eIF2α kinase necessary for activating eIF2α kinase (Guo et al., 2020). Another investigation further uncovered the OMA1-DELE1-HRI signaling axis that constitutes a link between mitochondrial perturbation and the cytosolic ISR. Moreover, this research combined genome engineering and haploid genetics to identify a suite of additional regulators in ISR. Therefore, this pathway is a potential therapeutic target, which can fine-tune ISR and obtain beneficial results in treating diseases involving mitochondrial dysfunction (Fessler et al., 2020). The serine/threonine kinase mTOR integrates extracellular and intracellular signals to drive growth and proliferation. mTORC1 is an important upstream regulator of integrated mitochondrial stress response (ISRmt). Khan et al. (2017) reported that mTORC1 is activated by mitochondrial DNA replication defects, which drive ISRmt through ATF4 activation. mTORC1 activation induces the mitochondrial one-carbon cycle, fibroblast growth factor 21 (FGF21), and the UPRmt. Downregulation of this response by rapamycin reverts progression of mitochondrial myopathy in mice (Khan et al., 2017). In mammals, mTOR coordinates the energy consumption of mRNA translation mechanisms with mitochondrial energy production by stimulating the synthesis of nuclear-encoded mitochondrial-related proteins, including mitochondrial transcription factor A (TFAM), mitochondrial ribosomal protein, and components of complexes I and V (Chapman et al., 2018). We have shown that mTOR, which is the upstream regulator of mitochondria, can sense mitochondrial translation defects and subsequently activate cytoplasmic translation to compensate for them (Gao et al., 2016). We recently found that mTOR adapts cytoplasmic translation to mitochondrial translation defects caused by mtEF4 ablation in *C. elegans* and mice (Gao et al., 2016) (Figure 2). Another example of mTOR regulation by mitochondrial stress is the ubiquitination of mTOR by Parkin, an E3 ubiquitin ligase located in mitochondria. Parkin is required to maintain mTORC1 activity during mitochondrial stress (Park et al., 2014). Thus, mTOR may be important in sensing mitochondrial translation defects and subsequently activating cytoplasmic translation. It is conceivable that cytoplasmic translation regulated by mTOR and mitochondrial translation controlled by mitochondrial translation factors, such as mtEF4, have a bidirectional causal relationship (Gao et al., 2016).

RNA interference to reduce expression of mitochondrial ribosomal protein MRPS5 leads to an imbalance of OXPHOS subunits (mitonuclear protein imbalance) encoded by nDNA and mtDNA. Expression of the mtDNA-encoded MT-CO1 homolog MTCE.26 and nDNA-encoded ATP5PF homolog H28O16.1 can be unbalanced (Houtkooper et al., 2013). In mammalian cells, MRP knockout results in mitochondrial protein imbalance, decreased mitochondrial respiration, and activation of the UPRmt. Specific antibiotics or ethidium bromide, targeting mitochondrial translation, can extend lifespan by inducing mitonuclear protein imbalance (Houtkooper et al., 2013). Silencing miRNA-382-5p significantly increases the expression of genes related to mitochondrial dynamics and biogenesis. Conventional microarray analysis revealed the downregulation of MRPs and respiratory chain proteins in C2C12 myotubes upon silencing of miRNA-382-5p. This effect was accompanied by an imbalance between mitochondrial proteins encoded by nDNA and mtDNA and induction of heat shock protein 60 (HSP60), indicating that UPRmt was activated and that silencing of miR-382-5p resulted in mitonuclear protein imbalance and activated UPRmt in skeletal muscle (Dahlmans et al., 2019). Chloramphenicol and other antibiotics inhibit mitochondrial protein translation, effectively decreasing the synthesis of mitochondrial proteins in INS-1E cells and reducing the expression of mtDNA encoding the COX1 subunit of the respiratory chain, rather than the ATP synthase subunit ATP5PF. Although expression of the important respiratory chain subunit COX1 was significantly reduced, the INS-1E cells maintained a normal respiratory rate, indicating that inhibition of mitochondrial protein translation caused mitonuclear protein imbalance. However, in insulin-secreting cells, a compensatory mechanism effectively maintained a normal respiratory rate and even increased ATP synthase-dependent respiration and calcium signaling pathways (Santo-Domingo et al., 2017).

## Conclusion

Mitochondria participate in important life activities of cells and are important in the study of evolution. Mitochondrial protein translation is an important and unique mitochondrial function, essential for the biogenesis of mitochondrial OXPHOS, cellular energy supply, and other mitochondrial functions. Mitochondrial protein translation is directly regulated by mitochondrial translation initiation, elongation, termination factors, translation activators, and mitomiRs. Mitochondrial translation and cytoplasmic translation are regulated by mTOR and other signaling pathways. Mitochondrial translation defects are the main causes of devastating human diseases. In this group of diseases, most mutations in mtDNA-encoding tRNAs, as well as mutations in nuclear genes encoding mitoribosomal proteins, translation initiation factors, and elongation factors, are the causes of clinical and genetic heterogeneity of infant multisystem diseases, such as Leigh syndrome, sensorineural hearing loss, encephalomyopathy, and hypertrophic cardiomyopathy (Perez-Martinez et al., 2008; Rotig, 2011). Many patients with mitochondrial diseases have multiple OXPHOS defects of unknown genetic causes, which indicates that many genes related to the biogenesis and function of mitochondrial translation mechanisms still need to be identified. Comprehensive clinical diagnosis and treatment based on mitochondrial translation defects remain challenging. With the advent of a boom in mitochondrial research and the progress in modern sequencing technology, the study of protein function will be more in-depth, and research on mitochondrial protein translation regulation will increase in importance. Research on mitochondrial protein translation will enhance our understanding of the pathogenesis and early diagnosis of human diseases.

## Author Contributions

YG conceived of the manuscript. YG, FW, DYZ, DJZ, and PL drafted the manuscript, constructed the figures, and revised the manuscript. All authors read and approved the final manuscript.

## Conflict of Interest

The authors declare that the research was conducted in the absence of any commercial or financial relationships that could be construed as a potential conflict of interest.

## Abbreviations

AD: Alzheimer’s disease
aa-tRNA: aminoacyl-tRNA
CTD: C-terminal domain
COXPD7: OXPHOS deficiency type 7
CPEO: chronic progressive external ophthalmoplegia
CRC: colorectal carcinoma
CTEs: mito-specific C-terminal extensions
MT-CYB: mitochondrial cytochrome b
EMT: epithelial-to-mesenchymal transition
ETS: electron transport system
fMet: formyl-methionine
GDP: guanosine diphosphate
GGQ: Gly-Gly-Gln
GTP: guanosine triphosphate
GTPBP6: GTP binding protein 6
GUF1: GTPase of unknown function 1
HUPRA syndrome: hyperuricemia, pulmonary hypertension, renal failure in infancy, and alkalosis
ISCU: iron-sulfur cluster assembly enzyme
ISR: integrated stress response
LBSL: leukoencephalopathy with brainstem and spinal cord involvement and lactate elevation
LRPPRC: leucine rich pentatricopeptide repeat containing
ISR: integrated stress response
LSU: large subunit
LTBL: leukoencephalopathy with thalamus and brainstem involvement and high lactate
MELAS: mitochondrial encephalomyopathy, lactic acidosis, and stroke-like episodes
MERRF: myoclonic epilepsy with ragged-red fiber
MIDD: maternally inherited diabetes and deafness
miRNA: microRNA
mitomiRs: mitochondrial miRNAs
MITRAC: mitochondrial translation regulation assembly intermediate of cytochrome c oxidase
MLASA, syndrome: myopathy, lactic acidosis, and sideroblastic anemia
mt-tRNA: mitochondrial transfer RNA
MT-TI: mt-tRNA^Ile^
MT-TL1: mt-tRNA^Leu^
MT-TK: mt-tRNA^Lys^
mt-aaRSs: mitochondrial aminoacyl-tRNA synthetases
mitoribosomes: mitochondrial ribosomes
mRNA: messenger RNA
mtDNA: mitochondrial DNA
mtPIC: mitochondrial preinitiation step
mTOR: mammalian target of rapamycin
MTRF: mitochondrial release factor
nDNA: nuclear DNA
NGS: next-generation sequencing
NTD: N-terminal domain
NTEs: mito-specific N-terminal extensions
OXPHOS: oxidative phosphorylation
PD: Parkinson’s disease
PoTC: post-termination complex
PTC: peptidyltransferase center
RCC: respiratory chain complexes
ROS: reactive oxygen species
RRF: mitochondrial ribosome recycling factor
rRNA: ribosomal RNA
SNP: single nucleotide polymorphism
SRL: sarcin/ricin loop
SSU: small subunit
TACO1: translational activator of cytochrome oxidase I
TFAM: mitochondrial transcription factor A
tRNA: transfer RNA
UPRmt: mitochondrial unfolded protein response
USP5: ubiquitin-specific peptidase 5
UTR: untranslated region
WES: whole-exome sequencing.
